# Supplementary material for: Health Equity Implications of the COVID-19 Lockdown and Visitation Strategies in Long-Term Care Homes in Ontario: A Mixed Method Study
Source: Int J Environ Res Public Health. 2022 Apr 2;19(7):4275. doi: 10.3390/ijerph19074275 (PMC8998692; doi:10.3390/ijerph19074275)
Supplement: Supplementary file 1 [file ijerph-19-04275-s001.zip › Supplementary material S1.pdf]

Supplementary material S1. Survey guide

1. My name is (please write your full name) ____________

2. My age is: _______ years

3. My gender is:
    1. Male
    2. Female
    3. Other

4. My preferred language of communication is:
    1. English
    2. French
    3. Other (please specify): ___________

5. My country of birth is:
    1. Canada
    2. Foreign born, please specify _________

6. I am:
    a. A LTC home resident
    b. A family/relative of an LTC resident
    c. A member of Resident associations/patient partnerships
    d. A provider of healthcare (both clinical and managerial)
    e. A policymaker
    f. A principal investigator and or a part of a research team
    g. A content expert/non-profit organization
    h. Medical student
    i. Nursing student
    j. Other (please specify):________

Section B. About visitation during the pandemic

7. (Ask if 6 = a) How often were you visited in-person/via audio call /via video call by your family or friends during the COVID-19 pandemic?

    1. Almost daily (question 14)

2. Once in two weeks (question 14)
3. Once in a month (question 14)
4. Never (question 15)
5. Others, please specify__________________ (question 14)

8. (Ask if 6 =b) How often have you visited (in person/ via audio call/video call) your loved ones residing in the long-term care institution?]

1. Almost daily (question 14)
2. Once in two weeks (question 14)
3. Once in a month (question 14)
4. Never (question 15)
5. Others, please specify__________________ (question 14)

9. (Ask if 7/ 8 is not equal to 4) Please tell us about your experience with those visitations. (open ended)

10. Now, I would like to know your perception about visitation strategies/interventions that can be implemented during a situation like COVID-19.

(All optional)

	10. Should this intervention be a priority for a LTC facility?	11. Is this intervention acceptable to you?	12. Do you think this intervention can be easily implemented in nursing homes/LTC?
A. Virtual visits (via Skype, Facetime, Duo, Zoom, What's App)	Participants can respond with:<br><br>1. Yes<br>2. Probably yes		

<table>
<tr><td>B. Window visits</td><td rowspan="5">3. Probably no
4. No

If participant choses options 3 or 4 for question 12, ask:
Why do you think it is not possible or probably not possible to implement this strategy?</td></tr>
<tr><td>C. Audio/video recorded messages</td></tr>
<tr><td>D. Family member visits with appropriate PPE* as a designated caregiver</td></tr>
<tr><td>E. Staff reading to residents printed out email messages received from families</td></tr>
<tr><td>F. Outdoor visits</td></tr>
</table>

<table>
<tr><th></th><th>13. What should be the duration of this visit (in minutes)?</th><th>14. What should be the frequency of such visits in a month?</th><th>15. How many visitors should be allowed per month for such visits?</th></tr>
<tr><td>A. Virtual visits (via Skype, Facetime, Duo, Zoom, What's App)</td><td rowspan="4">This is an open-ended question. Participants respond as they see fit.</td><td rowspan="4">Participants can respond with:
1. Once a month
2. Twice a month
3. Thrice a month
4. Daily
5. Other, please specify: -------------</td><td rowspan="4">This is an open-ended question. Participants respond as they see fit.</td></tr>
<tr><td>B. Window visits</td></tr>
<tr><td>C. Audio/video recorded messages</td></tr>
<tr><td>D. Family member visits with appropriate PPE* as a designated caregiver</td></tr>
</table>

E. Staff reading to residents printed out email messages received from families			
F. Outdoor visits			
