# Supplementary material for: Health Equity Implications of the COVID-19 Lockdown and Visitation Strategies in Long-Term Care Homes in Ontario: A Mixed Method Study
Source: Int J Environ Res Public Health. 2022 Apr 2;19(7):4275. doi: 10.3390/ijerph19074275 (PMC8998692; doi:10.3390/ijerph19074275)
Supplement: Supplementary file 1 [file ijerph-19-04275-s001.zip › Supplementary material S2.pdf]

Supplementary material S2. Interview guide

**Interviewer's initials:** _ _<br><br>**Participant's ID**: _ _ _ [For use by the central research unit only]<br><br>**Date of interview**: MM/DD/YYYY<br><br>**Interview medium (Please check ONE):**<br>❏ Video conferencing<br>❏ Phone call
**Participant's stakeholder group**:<br>❏ LTC staff/ provider of care<br>❏ LTC resident<br>❏ LTC resident's family member (designated caregiver)<br>❏ Other: Please specify ___________________
**Recording:**<br>Make sure you are recording the interview and using Otter for transcription
**Introduction**:<br>Hello, My name is _______ and I am working on a study examining the perspectives of different stakeholders regarding visitation strategies to long-term care homes in Ontario.
**Obtaining Informed Consent:**<br>➔ Please explain the nature and objectives of the study to the participant.<br><br>➔ If the participant has any questions, please answer them to the best of your knowledge.<br><br>➔ Ensure that the participant is aware of his/her rights to refuse answering any question or terminate the interview at his discretion and anytime.<br><br>➔ Emphasize the participant's privacy and confidentiality and describe the process of de-identifying his/her response from his/her name and other identifiers.<br><br>➔ Explain that the interview is being recorded and only research staff will have authority to view the recordings. Describe the method of safe keeping the recordings until termination

➔ Ensure the participant is aware of whom to contact to gain more information or report any issues or concerns.

➔ Acquire the informed consent. If informed consent was acquired, please proceed to the interview.

Interview questions:

1. Tell me how changes to visitation strategies have impacted [your work/ your relationship with your family member/ quality of life]. (pick the one most relevant to participant)

2. Can you tell me a story regarding your experience with visitation to long-term care homes during COVID-19?

   Now let's talk about these visitation strategies and quality of life in more detail.

3. In the survey, you answered questions about "designated caregivers" or **family member visits with appropriate personal protective equipment**. Tell me more about your response? [Probe for understanding of their experience]

   <u>Follow-up to question 3</u>: Please tell me why you think family member visits with appropriate personal protective equipment should be [this long/ this frequent/ with this many visitors]? [Probe for more stories]

4. In the survey, you have answered questions about **virtual visits**. Tell me more about your response?

   <u>Follow-up to question 4</u>: Please tell me why you think virtual visits should be [this long/ this frequent/ with this many visitors]?

5. Do you have any comments or stories about window visits that you would like to share?

6. Do you have any comments or stories about audio or video recorded messages?

7. Do you have any comments or stories about staff reading email messages received from families?

8. Do you have any comments or stories about outdoor visits?<br><br>9. Are there any **other visitation stories or comments** you would like to share?
**Thank the participant for participating in the interview. End the recording and Otter.**
