# Supplementary material for: Health Equity Implications of the COVID-19 Lockdown and Visitation Strategies in Long-Term Care Homes in Ontario: A Mixed Method Study
Source: Int J Environ Res Public Health. 2022 Apr 2;19(7):4275. doi: 10.3390/ijerph19074275 (PMC8998692; doi:10.3390/ijerph19074275)
Supplement: Supplementary file 1 [file ijerph-19-04275-s001.zip › Supplementary material S3.pdf]

Supplementary material S3. Raw quantitative results from the survey (N=201)

Visitation strategy	Essential caregivers		Outdoor visits		Window visits		Virtual visits		Audio/ video recorded messages		Printed emails read by staff	
	n	%	n	%	n	%	n	%	n	%	n	%
**Priority**												
**Yes**	147	73.1%	117	58.2%	89	44.3%	127	63.2%	39	19.4%	80	39.8%
**Probably Yes**	35	17.4%	49	24.4%	60	29.9%	55	27.4%	62	30.8%	69	34.3%
**Probably No**	9	4.5%	21	10.4%	36	17.9%	11	5.4%	64	31.8%	38	18.9%
**No**	6	3.0%	8	4.0%	16	8.0%	7	3.5%	34	16.9%	9	4.5%
**Missing***	4	2.0%	6	3.0%	0	0.0%	1	0.5%	2	1.0%	5	2.5%
**Feasibility**												
**Yes**	106	52.7%	89	44.3%	85	42.3%	120	59.7%	58	28.9%	96	47.8%
**Probably Yes**	56	27.9%	67	33.3%	59	29.4%	54	26.9%	75	37.3%	53	26.4%
**Probably No**	29	14.4%	30	14.9%	36	17.9%	20	10.0%	45	22.4%	36	17.9%
**No**	6	3.0%	9	4.5%	21	10.4%	7	3.5%	22	10.9%	12	6.0%
**Missing***	4	2.0%	6	3.0%	0	0.0%	0	0.0%	1	0.5%	4	2.0%
**Acceptability**												
**Yes**	143	71.1%	135	67.2%	103	51.2%	132	65.7%	71	35.3%	109	54.2%
**Probably Yes**	38	18.9%	38	18.9%	40	19.9%	28	13.9%	47	23.4%	42	20.9%
**Probably No**	13	6.5%	13	6.5%	26	12.9%	19	9.5%	45	22.4%	27	13.4%
**No**	3	1.5%	9	4.5%	32	15.9%	22	10.9%	36	17.9%	19	9.5%
**Missing***	4	2.0%	6	3.0%	0	0.0%	0	0.0%	2	1.0%	4	2.0%

* Participants had the option to not respond to questions. Their data was considered missing
