# Supplementary material for: Health Equity Implications of the COVID-19 Lockdown and Visitation Strategies in Long-Term Care Homes in Ontario: A Mixed Method Study
Source: Int J Environ Res Public Health. 2022 Apr 2;19(7):4275. doi: 10.3390/ijerph19074275 (PMC8998692; doi:10.3390/ijerph19074275)
Supplement: Supplementary file 1 [file ijerph-19-04275-s001.zip › Supplementary material S4.pdf]

Supplementary material S4. Participants' responses to questions about the duration and frequency of visits and number of visitors allowed

Table 1. Survey responses (N=201) regarding the duration of visits to long-term care homes

Duration of visits	Essential caregivers		Outdoor visits		Window visits		Virtual visits		Audio/ video recorded messages		Printed emails read by staff	
	n	%	n	%	n	%	n	%	n	%	n	%
Less than 30 min	32	15.9%	89	44.3%	94	46.8%	123	61.2%	105	52.2%	113	56.2%
30-60 mins	50	24.9%	38	18.9%	11	5.5%	8	4.0%	3	1.5%	0	0.0%
Over 60 mins	22	10.9%	4	2.0%	0	0.0%	0	0.0%	0	0.0%	0	0.0%
Unlimited	37	18.4%	5	2.5%	9	4.5%	3	1.5%	6	3.0%	4	2.0%
Other*	19	9.5%	15	7.5%	25	12.4%	24	11.9%	10	5.0%	22	10.9%
Missing**	41	20.4%	50	24.9%	62	30.8%	43	21.4%	77	38.3%	62	30.8%

Responses categorized during analysis to: less than 30 minutes, 30-60 minutes, over 60 minutes, unlimited duration, other, and missing responses

* Other: Participants were given the option to answer this question with an open-ended box, analyzed qualitatively

** Participants had the option not to respond to any question. Their data was considered missing

Table 2. Survey responses (N=201) regarding the frequency of visitation strategies to long-term care homes

Frequency of visits	Essential caregivers		Outdoor visits		Window visits		Virtual visits		Audio/ video recorded messages		Printed emails read by staff	
	n	%	n	%	n	%	n	%	n	%	n	%
Daily	84	41.8%	42	20.9%	36	17.9%	38	18.9%	31	15.4%	44	21.9%
Once/ month	0	0.0%	3	1.5%	3	1.5%	0	0.0%	6	3.0%	8	4.0%
Twice/ month	9	4.5%	15	7.5%	13	6.5%	10	5.0%	14	7.0%	13	6.5%
Thrice/ month	23	11.4%	30	14.9%	23	11.4%	34	16.9%	26	12.9%	26	12.9%

**Other ***	46	22.9%	65	32.3%	68	33.8%	92	45.8%	56	27.9%	57	28.4%
**Missing ****	39	19.4%	46	22.9%	58	28.9%	27	13.4%	68	33.8%	53	26.4%

ponses categorized before analysis to: daily visits, once a month, twice a month, thrice a month, other, and missing responses
* Other: Participants were given the option to answer this question with an open-ended box, analyzed qualitatively
** Participants had the option not to respond to any question. Their data was considered missing

Table 3. Survey responses (N=201) on the number of visitors allowed for visitation strategies to long-term care homes

**Number of visitors**	**Essential caregivers**		**Outdoor visits**		**Window visits**		**Virtual visits**		**Audio/ video recorded messages**		**Printed emails read by staff**	
	**n**	**%**	**n**	**%**	**n**	**%**	**n**	**%**	**n**	**%**	**n**	**%**
**1 to 3 visitors**	96	47.8%	62	30.8%	29	14.4%	35	17.4%	28	13.9%	31	15.4%
**4 to 8 visitors**	17	8.5%	38	18.9%	36	17.9%	37	18.4%	24	11.9%	35	17.4%
**> 8 visitors**	3	1.5%	8	4.0%	6	3.0%	10	5.0%	11	5.5%	5	2.5%
**Unlimited**	10	5.0%	14	7.0%	16	8.0%	21	10.4%	23	11.4%	25	12.4%
**Other***	36	17.9%	24	11.9%	50	24.9%	64	31.8%	26	12.9%	29	14.4%
**Missing****	39	19.4%	55	27.4%	64	31.8%	34	16.9%	89	44.3%	76	37.8%

* Other: Participants were given the option to answer this question with an open-ended box, analyzed qualitatively
** Participants had the option not to respond to any question. Their data was considered missing

Table 4. Qualitative findings from open-ended survey comments and one-on-one interviews

Subthemes:

➢ Participants commented that the duration and frequency of visits and number of visitors allowed are dependent on the needs of long-term care residents, the roles and responsibilities of visitors, and circumstance of the family.

➢ Many participants believed that designated caregivers should have unlimited access to their loved ones in long-term care homes

- While many participants provided specific responses to how long and how frequent a visit should be and how many visitors should be allowed, the majority specified that these responses are specific to their context and family circumstances

Quotes:

"*... one to two hours [duration], I thought was best. But again, that would probably be changed, given on what you may do in those two hours. So it may be longer depending on what you do for them*" (Interview B)

"*I think it [frequency of visits] depends on the circumstances. For myself, I am absolutely content with once a week, but other people need more than that*" (Interview C)

"*It [number of visitors] depends on the family ... some of those people have 20 grandchildren, and they want to see them all right. And some only have one child ... I think it depends*" (Interview J)
